# Possible indication of endoscopic resection in undifferentiated early gastric cancer

**DOI:** 10.1038/s41598-019-53374-0

**Published:** 2019-11-14

**Authors:** Dae Gon Ryu, Cheol Woong Choi, Su Jin Kim, Dae Hwan Kang, Hyung Wook Kim, Su Bum Park, Hyeong Seok Nam

**Affiliations:** Department of Internal Medicine, Pusan National University School of Medicine and Research Institute for Convergence of Biomedical Science and Technology, Pusan National University Yangsan Hospital, Yangsan, Korea

**Keywords:** Oesophagogastroscopy, Gastric cancer

## Abstract

Endoscopic resection for early gastric cancer (EGC) without lymph node metastasis may be a valuable treatment option. To date, endoscopic resection for undifferentiated EGC is being investigated. We evaluated the risk of lymph node metastasis in undifferentiated EGC by examining the preoperative endoscopic findings and operated pathologic specimen. The medical records of patients who underwent surgical resection because of undifferentiated EGC between November 2008 and December 2015 were reviewed retrospectively. The risk factors associated with lymph node metastasis and the lymph node metastasis rate in the expanded indication of undifferentiated EGC were evaluated. A total of 376 patients with undifferentiated EGC (233 signet ring cell type and 143 poorly differentiated type) were analyzed. Lymph node metastasis was found in 9.8% of the patients. Among the patients who met the expanded criteria (59 patients), only one patient had lymph node metastasis (signet ring cell type without ulceration and 15 mm in size). The risk factors associated with lymph node metastasis were lesion size >20 mm (OR 3.013), scar deformity (OR 2.248), surface depression (OR 2.360), submucosal invasion (OR 3.427), and lymphovascular invasion (OR 6.296). Before endoscopic resection of undifferentiated EGC, careful selection of patients should be considered. The undifferentiated EGC with size ≥15 mm, scar deformity, surface depression, submucosal invasion, and lymphovascular invasion should be considered surgical resection instead of endoscopic resection.

## Introduction

Endoscopic resection for early gastric cancer (EGC) without lymph node metastasis may be a treatment of choice. Although definite prediction of lymph node metastasis is prerequisite before the decision of endoscopic resection, imaging examination such as abdominal computed tomography, positron emission tomography, or endoscopic ultrasound cannot reliably confirm or exclude the presence of lymph node metastasis since it is impossible to predict the status lymph node metastasis definitely^1^. A differentiated mucosal adenocarcinoma 20 mm or less in size without ulceration may be recommended for endoscopic resection, usually endoscopic submucosal dissection (ESD) by Japanese and Korean gastric cancer guidelines (so-called absolute indication)^2,3^. In South Korea, the gastric cancer screening rate is approximately 70%^4^. As the rate of gastric cancer screening increases, the detection rate of early gastric cancer is also increasing. According to the nationwide survey on surgically treated gastric cancer in South Korea, the EGCs accounted for 57.6% of all cases, and 62.6% of patients showed no lymph node metastasis^5^. As the techniques and endoscopic instruments evolved, experienced endoscopists have removed the EGC regardless of lesion size. Gotoda *et al*. reported that the lymph node metastasis of undifferentiated EGCs 20 mm or less without associated ulcer formation and lymphovascular invasion was absent^6^. Therefore, these lesions might be a candidate for endoscopic resection.

According to the Japanese gastric cancer treatment guidelines, differentiated carcinoma includes papillary and well to moderate tubular adenocarcinoma, and undifferentiated carcinoma includes poorly differentiated adenocarcinoma and signet-ring cell carcinoma^2^. Although many studies reported the feasibility of endoscopic resection for undifferentiated EGC, the *en bloc* resection and curative resection rate were 92.1% and 61.4%, respectively^7^. However, if the ESD for undifferentiated EGC could achieve curative resection, an excellent 5-year mortality (gastric cancer-related mortality 0%) had been reported^8^. However, regardless of favorable outcomes after endoscopic resection, many clinicians suspect about the safety to do ESD for undifferentiated type EGC.

In the present retrospective study, we investigated the risk of lymph node metastasis in undifferentiated EGC by examining the operated results of undifferentiated EGC. Moreover, the lymph node metastasis rate in the expanded criteria of undifferentiated type EGC was evaluated.

## Patients and Methods

**Figure 1:**
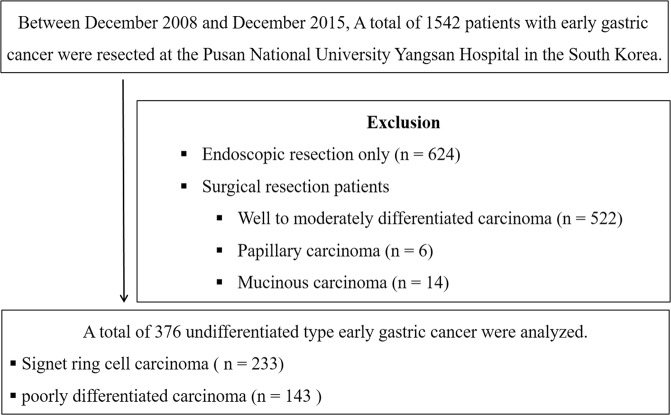
Study flow.

The medical records of patients who underwent resection because of gastric adenocarcinoma between November 2008 and December 2015 were reviewed retrospectively at the Pusan National University Yangsan Hospital in South Korea. During the study period, a total of 1542 patients diagnosed with EGC underwent resection (included 624 endoscopic resection). Among the patients who underwent surgical resection, those with well to moderately differentiated carcinoma (n = 522), papillary carcinoma (n = 6), and mucinous carcinoma (n = 14) were excluded. Finally, a total of 376 patients with undifferentiated type EGC who underwent surgical resection were enrolled and analyzed (233 signet ring cell type carcinoma and 143 poorly differentiated carcinoma) (Fig. 1). The study was approved by the ethics committee of the Pusan National University Yangsan Hospital review board (Institutional Review Board no. 05-2018-157). And informed consent was waived because the subject’s medical records were anonymized before analysis. The study was conducted in accordance with the principles of the Declaration of Helsinki. There were no conflicts of interest or sponsors in this study.

### Procedure and endoscopic examination

In general practice at our institution, for patients with EGC without evidence of lymph node metastasis by abdominal computed tomography, laparoscopic gastrectomy with D1+ lymph node dissection was performed according to the Japanese gastric cancer treatment guidelines 2014 (ver. 4)^2^. The day before the surgery, endoscopic metal clips were applied just 2 cm proximal to the site of EGC. During endoscopic examinations, we evaluated the endoscopic characteristics of EGC (location of lesion, gross type, and surface configuration of EGC).

### Clinicopathologic factors and definitions

**Figure 2:**
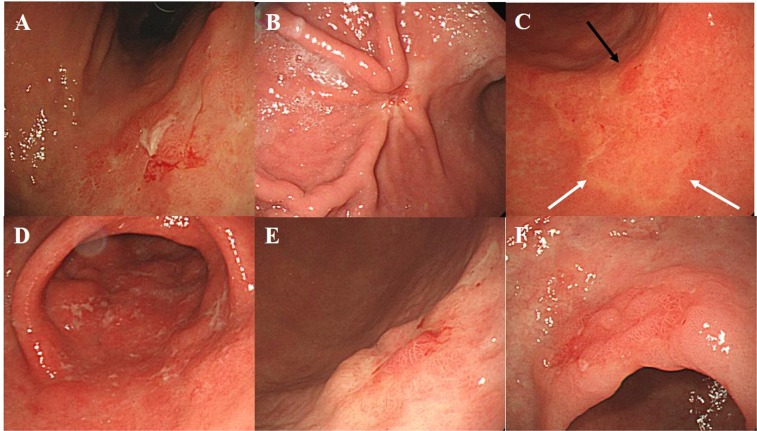
Surface configuration of undifferentiated type early gastric cancer. (**A**) Active ulceration, (**B**). Scarring deformity, (**C**). erythema (black arrow) and discoloration (white arrow), (**D**). Surface erosions with white exudate, (**E**). central depression at the elevated type early gastric cancer, (**F**). nodular surface appearance.

For each patient, the baseline characteristics and endoscopic features were reassessed by two endoscopists (CW Choi, MD, PhD and DG Ryu, MD). The location of EGC was classified according to the Japanese classification of gastric cancer: upper, middle, and lower third of the stomach^9^. Endoscopic atrophic gastritis was diagnosed when visible vascular pattern in the submucosa under pale mucosa was observed. The extent of endoscopic atrophic gastritis was classified according to the Kimura and Takemoto classification: mild (normal to closed type 2), moderate (closed type 3 to open type 1), or severe (open type 2 to open type 3)^10^. Gross appearance of EGC was classified according to the Paris classification of superficial gastric neoplastic lesions^11^: elevated (0-Ip, 0-Is, or 0-IIa), flat (0-IIb) or depressed (0-IIc or 0-III). After determining the location and gross appearance of EGC, surface configurations were described. If active excavated ulcer or scarring deformity secondary to the previous ulceration (converging fold or deformity) occurs, the finding was regarded as ulcer-related lesion. Surface erythema or discoloration was classified after comparing it to the surrounding non-cancerous mucosa. A reddish color change was regarded as erythema, and a discoloration was regarded as fading color change. Surface nodularity was the presence of irregularly raised mucosa. Erosion was defined as acute flat defect of the mucosa characterized as movable and round or linear mucosal defect not exceeding a few millimeters upon endoscopic forceps biopsy^12^. Surface depression was defined as the central depression of the tumor surface compared to the height of the EGC regardless of ulceration (0-IIa + IIc, 0-IIb + IIc or 0-IIc + III) (Fig. 2). The two endoscopists were trained to review about 100 typical endoscopic photographs before evaluating the endoscopic biopsy images. Of the 376 lesions, the diagnoses were consistent for 342 lesions between the two endoscopists. For 34 lesions that differed in opinion, the final diagnosis was made through discussion and consensus.

Surgically resected tissue slides were reviewed by two different pathologists, who were blinded to patient’s information. Discordant diagnosis were re-evaluated through a multi-headed microscope to attain a consensus.

The pathologic type of EGC, lesion size, submucosal invasion, lymphovascular invasion, and perineural invasion were evaluated. The pathologic type of undifferentiated type EGC was classified as signet ring cell type and poorly differentiated type EGC. If different histologic components were found in one surgical specimen, the histologic type of EGC was classified according to the predominant histologic type.

### Statistical analysis

Statistical Package for the Social Sciences (SPSS) version 21.0 (IBM Corp., Armonk, NY, USA) was used for the statistical analyses. Univariate analysis was performed using chi-square test or Fisher’s exact test. Multivariate analysis was performed using multiple logistic regression models in analyzing associated factors for lymph node metastasis. A *p* < 0.05 was considered statistically significant.

### Ethical standard

The study was approved by the ethics committee of the Institutional Review Board of Pusan National University Yangsan Hospital (Institutional Review Board No. 05-2018-157). There were no conflicts of interest or sponsors in this study.

## Results

Baseline characteristics of patients were summarized in Table 1. During the study period, a total of 376 patients with undifferentiated EGC (233 signet ring cell type and 143 poorly differentiated type) were analyzed. Mean patients age was 60.2 ± 12.1 years, and 52.9% of the patient cohort was male. The predominant location of lesion was the middle third of the stomach (51.6%). The mean tumor size was 32.4 ± 19.7 mm. Moderate extent of endoscopic atrophic gastritis was predominant (55.1%). The most common gross type was depressed (76.8%) followed by flat (16.2%) and elevated (6.9%). The endoscopic findings about the surface configuration were shown in Table 1: excavated ulceration (40.2%), scar deformity (41.2%), erythema (93.1%), discoloration (24.2%), nodularity (22.6%), depression (33.8%), and erosion (33.0%). Submucosal cancer invasion was found in 42.0% of the patients. Further, lymphovascular and perineural invasions were found in 7.4% and 5.9% of patients, respectively.

**Table 1:** Baseline characteristics.

	Total (n = 376)
Age, years, mean (SD)	60.2 (12.1)
Male gender, n (%)	199 (52.9)
**Tumor location, n (%)**	
Lower third	143 (38.0)
Middle third	194 (51.6)
Upper third	39 (10.4)
Lesion size, mm, mean (SD)	32.4 (19.7)
Signet ring cell type, n (%)	233 (62.0)
**Atrophic gastritis, n (%)**	
Mild	118 (31.4)
Moderate	207 (55.1)
Severe	51 (13.6)
**Gross type, n (%)**	
Elevated	26 (6.9)
Flat	61 (16.2)
Depressed	289 (76.9)
**Surface configuration, n (%)**	
Excavated ulceration	151 (40.2)
Scar deformity	155 (41.2)
Ulcer-related findings	246 (65.4)
Erythema	350 (93.1)
Discoloration	91 (24.2)
Nodularity	85 (22.6)
Depression	127 (33.8)
Erosion	124 (33.0)
**Pathologic factors**	
Submucosal invasion, n (%)	158 (42.0)
Lymphovascular invasion, n (%)	28 (7.4)
Perineural invasion, n (%)	22 (5.9)
Depth of invasion Signet ring cell type: M1/M2/SM1/deep SM, n (%)	93/64/10/66
	(39.9/27.5/4.3/28.3)
Poorly differentiated type: M1/M2/SM1/deep SM, n (%)	31/30/11/71
	(21.7/21.0/7.7/49.6)
Within expanded indication, n (%)	59 (15.7)
Lymph node metastasis, n (%)	37 (9.8)

SD, standard deviation; SM, submucosa; M1, lamina propria; M2, muscularis mucosa; SM1, <500 μm from the muscularis mucosa; deep SM, >500 μm from the muscularis mucosa.

Comparative analysis to know the risk factors associated with lymph node metastasis was performed. By univariate analysis, lesion size >20 mm, excavated ulceration, scar deformity, nodularity submucosal invasion, lymphovascular invasion, and perineural invasion were significant (Table 2). By multivariate analysis, lesion size >20 mm (OR 3.013, 95% CI 1.029–8.825, p = 0.044), scar deformity (OR 2.248, 95% CI 1.006–5.021, p = 0.048), surface depression (OR 2.360, 95% CI 1.051–5.301, p = 0.037), submucosal invasion (OR 3.427, 95% CI 1.309–8.971, p = 0.012), and lymphovascular invasion (OR 6.296, 95% CI 2.414–16.421, p < 0.001) were significant (Table 3).

**Table 2:** Risk factors analysis associated with lymph node metastasis in the undifferentiated type early gastric cancer; univariate analysis

	Absence of lymph node metastasis (n = 339)	Presence of lymph node metastasis (n = 37)	Total (n = 376)	P value
Age, years, mean (SD)	60.2 (11.9)	59.8 (14.5)	60.2 (12.1)	0.842
Male gender, n (%)	179 (52.8)	20 (54.1)	199 (52.9)	0.885
**Tumor location, n (%)**				**0.568**
Lower third	126 (37.2)	17 (45.9)	143 (38.0)	
Middle third	177 (52.2)	17 (45.9)	194 (51.6)	
Upper third	36 (10.6)	3 (8.1)	39 (10.4)	
Lesion size, mm, mean (SD)	30.7 (18.4)	47.8 (25.1)	32.4 (19.7)	<0.001
Lesion size >20 mm, n (%)	210 (61.9)	32 (86.5)	242 (64.4)	0.003
Signet ring cell type, n (%)	214 (63.1)	19 (51.4)	233 (62.0)	0.161
**Atrophic gastritis, n (%)**				**0.434**
Mild	105 (31.0)	13 (35.1)	118 (31.4)	
Moderate	190 (56.0)	17 (45.9)	207 (55.1)	
Severe	44 (13.0)	7 (18.9)	51 (13.6)	
**Gross type, n (%)**				**0.437**
Elevated	22 (6.5)	4 (10.8)	26 (6.9)	
Flat	57 (16.8)	4 (10.8)	61 (16.2)	
Depressed	260 (76.7)	29 (78.4)	289 (76.9)	
**Surface configuration, n (%)**				
Excavated ulceration	130 (38.3)	21 (56.8)	151 (40.2)	0.030
Scar deformity	133 (39.2)	22 (59.5)	155 (41.2)	0.018
Ulcer-related findings	217 (64.0)	29 (78.4)	246 (65.4)	0.081
Erythema	313 (92.3)	37 (100)	350 (93.1)	0.081
Discoloration	82 (24.2)	9 (24.3)	91 (24.2)	0.985
Nodularity	71 (20.9)	14 (37.8)	85 (22.6)	0.020
Depression	105 (31.0)	22 (59.5)	127 (33.8)	0.001
Erosion	112 (33.0)	12 (32.4)	124 (33.0)	0.941
**Pathologic factors, n (%)**				
Submucosal invasion	128 (37.8)	30 (81.1)	158 (42.0)	<0.001
Lymphovascular invasion	14 (4.1)	14 (37.8)	28 (7.4)	<0.001
Perineural invasion	16 (4.7)	6 (16.2)	22 (5.9)	0.005
Within expanded indication*, n (%)	58 (17.1)	1 (0.3)	59 (15.7)	0.022

SD, standard deviation

*Undifferentiated type, early gastric cancer without ulceration limited in the mucosa, ≤20 mm.

**Table 3:** Risk factors analysis associated with lymph node metastasis in the undifferentiated type early gastric cancer; multivariate analysis.

	Odd ratio	95% Confidence interval	p value
Lesion size >20 mm,	3.013	1.029–8.825	0.044
Excavated ulceration	1.134	0.507–2.538	0.758
Scar deformity	2.248	1.006–5.021	0.048
Nodularity	1.241	0.539–2.855	0.611
Depression	2.360	1.051–5.301	0.037
Submucosal invasion, n (%)	3.427	1.309–8.971	0.012
Lymphovascular invasion, n (%)	6.296	2.414–16.421	<0.001
Perineural invasion, n (%)	1.668	0.520–5.349	0.389

**Figure 3:**
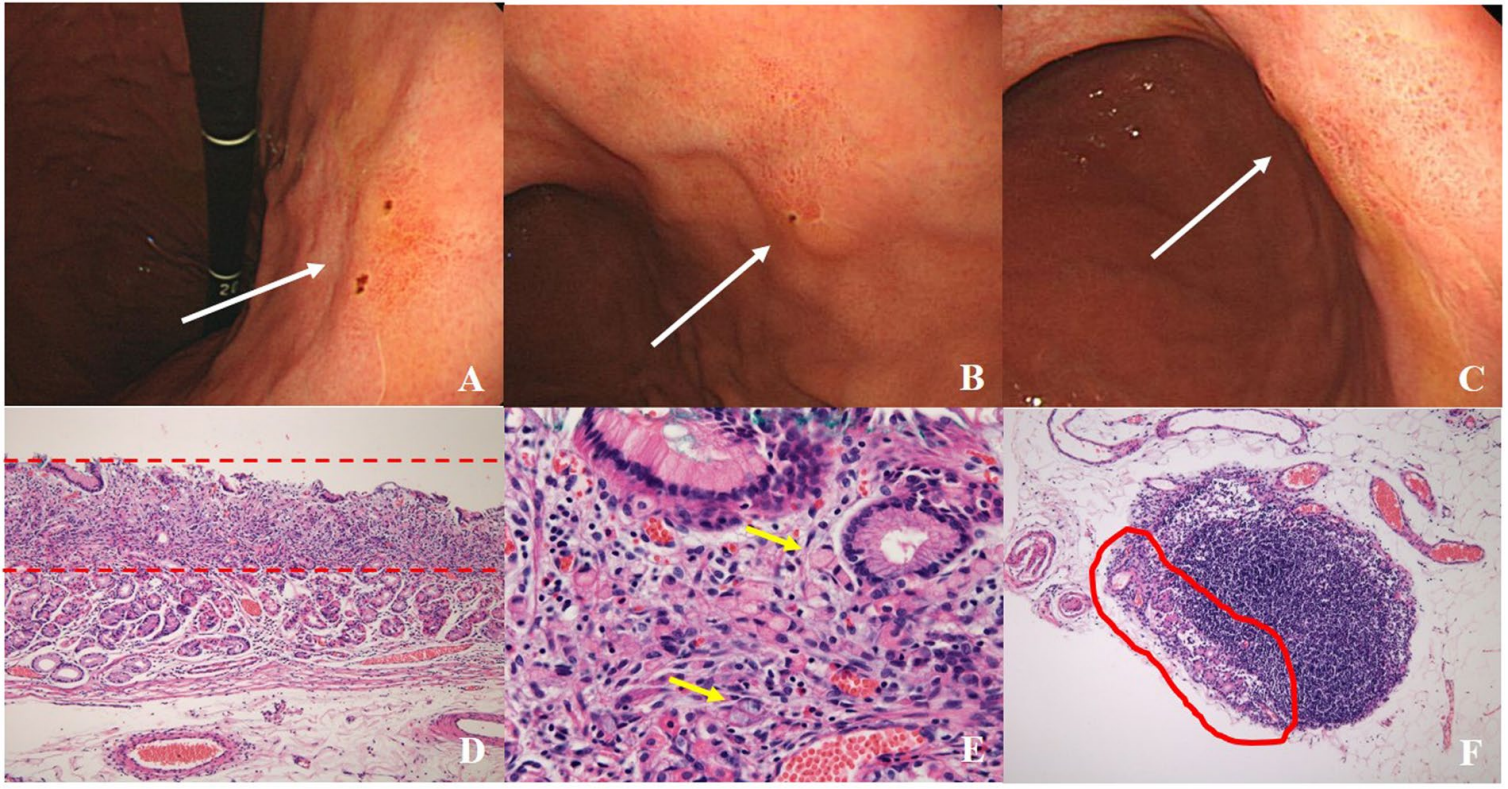
A case of lymph node metastasis of signet ring cell type early gastric cancer. (**A–C**) At the posterior wall of the lower body, a flat reddish mucosal lesion was found (white arrow). (**D,E**) After surgical resection, the lesion size was 15 * 15 mm, signet ring cell type early gastric cancer within lamina propria. (100× and 200×, respectively). Yellow arrow indicated signet ring cell type adenocarcinoma. (**F**) The lymph node metastasis was found. (100×).

Among enrolled patients, 59 (15.7%) had undifferentiated EGC without ulceration and lymphovascular invasion. Among these patients, only one patient (1.6%, 1/59) showed lymph node metastasis after surgical resection (Fig. 3).

## Discussion

An ESD has been the standard treatment of EGC for a differentiated type without ulceration, of which is limited in the mucosa and 20 mm or less in size (absolute indication for endoscopic resection)^2,3^. In recent years, EGCs diagnosed as (1) differentiated type EGC > 20 mm without ulceration and lymphovascular invasion, (2) differentiated type EGC ≤ 30 mm with ulceration, and (3) undifferentiated type EGC ≤ 20 mm without ulceration and lymphovascular invasion were regarded as the expanded investigational indication for endoscopic resection^2,3^. According to several studies comparing the clinical outcomes between absolute and expanded indication (excluding undifferentiated type EGC), the overall survival rate and relapse-free survival rate were similar^13–15^. However, the endoscopic treatment for undifferentiated type EGC has been an investigation indication. Previous studies in Japan reported that the incidence of lymph node metastasis of intramucosal undifferentiated gastric cancer 20 mm or less without lymphovascular invasion and ulcerative findings was zero^6,16^. In the present study, we analyzed the lymph node metastasis rate and associated risk factors of undifferentiated type EGC of patients who underwent surgical gastrectomy and lymph node dissection. The overall lymph node metastasis rate was 9.8% and the associated risk factors were lesion size >20 mm, scar deformity, surface depression, submucosal invasion, and lymphovascular invasion. The overall risk factors of lymph node metastasis were similar with the previous studies^6,16^. In the present study, among the 37 patients with EGCs who met the expanded criteria, one patient had lymph node metastasis (Fig. 3). The lesion was signet ring cell type EGC without surface ulceration and lymphovascular invasion (15 mm in size). Therefore, from the present study results, the lesion size which is considered for endoscopic resection should be less than 15 mm in size.

To do endoscopic resection for EGC, the precise prediction of lymph node metastasis is a prerequisite. However, the current role of imaging in assessing lymph node metastasis in gastric cancer cannot reliably confirm the presence of lymph node metastasis^1^. Therefore, to predict the possible status of lymph node metastasis, assessing the associated risk factors is considered important. We classified the risk factors as endoscopic factors (predictably by endoscopic examination such as lesion size, ulcer scar, and surface depression) and pathologic factors which can be determined after endoscopic resection (submucosal invasion and lymphovascular invasion).

To decide for endoscopic resection of undifferentiated EGC, the precise prediction of cancer margin is important. However, in undifferentiated EGC, endoscopic prediction of the lesion size and lateral margin may be somewhat different from the pathologic results. Although indigo carmine chromoendoscopy may enhance the discrimination of resection margin for differentiated EGC, its usefulness is reduced in undifferentiated EGC^17,18^. An image-enhanced endoscopy with magnification examining the microsurface and microvascular pattern on the mucosal surface is a useful to delineate the margins of differentiated EGC^19^. Undifferentiated EGCs were associated with false negative during image enhanced endoscopy with magnification^20^. The endoscopic detection of the undifferentiated EGC is difficult because undifferentiated intramucosal cancer sometimes spread within the mucosa beyond their macroscopic lateral margin^21^. The proliferative zone of undifferentiated intramucosal cancer was located in the intermediate zone of the mucosa among the 12.5% depressed type EGC and 85.7% flat type EGC^22^. Especially, tubule neck dysplasia is the possible precursor lesion of signet ring cell gastric cancer^23^. Therefore, when undifferentiated gastric cancer is suspected, a biopsy should be performed at the peripheral site of the lesion. This is also useful in assessing the lesion’s margin^21,22,24^.

Japanese investigators proposed the concept of a malignant cycle to explain the natural course of early gastric cancer^25^. According to the malignant cycle, at the initial stage of EGC, cancer cells might be removed from the mucosal surface through the action of gastric acid and pepsin^25^. Therefore, the active ulceration and ulcer scar deformity implied repeated healing and relapsing^25^. Hence, ulcer scar and surface depression of EGC may be associated with the repeated malignant ulcer cycles and are associated with the risk of lymph node metastasis.

The presence of lymphovascular invasion can be examined only by pathologic examination of the resected specimen. Endoscopic prediction of submucosal invasive cancer is important to decide for endoscopic resection. During conventional endoscopic examination, smooth surface protrusion or depression, erosion, and marginal elevation were known as endoscopic characteristics of EGC confined in the mucosa^26^. Irregular or nodular surface with or without abnormal converging folds (such as clubbing, abrupt cutting, and fusion) and deep ulceration with marked marginal elevation were acknowledged as characteristic endoscopic findings associated with submucosal invasive lesion^26^. However, the overall accuracy of endoscopic prediction of invasion depth for EGC was reported as 73–78%^26–28^. However, the accurate diagnosis rate was relatively lower in the undifferentiated EGC than in the differentiated EGC^26^. Endoscopic ultrasound is considered useful for predicting the invasion depth of gastrointestinal cancer. However, the overall accuracy of differentiating mucosal from submucosal invasive EGC was reported as 67.4–79.1^27,28^. Comprehensive accuracy combined with conventional endoscopy and endoscopic ultrasound may exceed up to 85%^28^. Therefore, several endoscopic findings to discriminate of invasion depth are not completely reliable.

Several studies reported on the endoscopic treatment outcomes of undifferentiated EGC. The overall *en bloc* resection and curative resection rate was 92.1% and 61.4%, respectively^7^. However, with the endoscopic treatment indication restricted in the expanded indication, the *en bloc* and curative resection rate improved up to 91.2% and 79.8%, respectively^7^. Although long-term follow-up data are limited, during 36–76.4 months’ follow-up, the overall mortality rate (gastric cancer-related death) and local recurrence rate after curative endoscopic resection of undifferentiated EGC were 0% and 0–4.2%, respectively^8,29,30^.

Signet ring cell carcinoma and poorly differentiated tubular adenocarcinomas are both classified as undifferentiated-type carcinomas. However, the pathophysiological progression of cancer differs between these. In particular, signet ring cell carcinoma shows intramucosal spreading^21,22,31^. For these reasons, there are reports the submucosal invasion and lymph node metastasis rate of signet ring cell type is lower than that of the other undifferentiated-type carcinomas^7,32–37^. In our study, also signet ring cell carcinoma also showed lower submucosal invasion and lymph node metastasis rate than that of poorly differentiated carcinoma (33.0% vs 56.6% in submucosal invasion, 8.6% vs 11.9% in lymph node metastasis).

The present study has several limitations. First, because of the retrospective study design analyzing the surgically resected specimen of undifferentiated EGC at a single academic hospital, selection bias may be inevitable. Second, when evaluating the endoscopic findings associated with EGC, image-enhanced endoscopy with magnification and endoscopic ultrasound might provide more valuable information associated with lymph node metastasis. However, previous several studies reported the inappropriate accuracy about the lymph node metastasis status and depth of invasion. Therefore, these endoscopic modalities are not indispensable modalities to decide treatment or not. Third, most patients were not tested for *Helicobacter* infection prior to surgery.

In summary, in the present study, the lymph node metastasis found in 9.8% of patients with undifferentiated EGC. However, among the patients who met the expanded criteria, the lymph node metastasis was found in only one patient (one among 59 patients, 1.6%). The established treatment for undifferentiated EGC is still gastrectomy with lymph node dissection^2^. But recently there are reports on good progress of endoscopic resection for undifferentiated EGC corresponding to expanded indication^3,8,16,29,30^. Our study also showed similar results, but lymph node metastasis was found in one patient with expanded criteria and the lesion size was 15 mm. Although endoscopic resection is considered for undifferentiated EGC within expanded indication, this option requires careful selection as lymph node metastasis is possible. In conclusion, when endoscopic treatment rather than surgery is considered for undifferentiated EGC, a multidisciplinary approaches with insight from an endoscopist, surgeon, radiologist, and pathologist is recommended. The undifferentiated EGC with size ≥15 mm, scar deformity, surface depression, submucosal invasion, and lymphovascular invasion should be considered surgical resection instead of endoscopic resection.
